# Flow-driven micro-scale pH variability affects the physiology of corals and coralline algae under ocean acidification

**DOI:** 10.1038/s41598-019-49044-w

**Published:** 2019-09-06

**Authors:** S. Comeau, C. E. Cornwall, C. A. Pupier, T. M. DeCarlo, C. Alessi, R. Trehern, M. T. McCulloch

**Affiliations:** 1 The University of Western Australia, Oceans Graduate School and Oceans Institute, 35 Stirling Highway, Crawley, 6009 Western Australia Australia; 2 ARC Centre of Excellence for Coral Reef Studies, 35 Stirling Highway, Crawley, 6009 Western Australia Australia; 3 Sorbonne Université, CNRS-INSU, Laboratoire d’Océanographie de Villefranche, 181 chemin du Lazaret, F–06230 Villefranche-sur-mer, France; 4 School of Biological Sciences, Victoria University of Wellington, Wellington, New Zealand; 5 Centre Scientifique de Monaco, 8 Quai Antoine Ier, MC-98000 Monaco, Monaco; 6 Sorbonne Université, Collège doctoral, F-75005 Paris, France

**Keywords:** Climate-change ecology, Environmental impact, Marine biology

## Abstract

Natural variability in pH in the diffusive boundary layer (DBL), the discrete layer of seawater between bulk seawater and the outer surface of organisms, could be an important factor determining the response of corals and coralline algae to ocean acidification (OA). Here, two corals with different morphologies and one coralline alga were maintained under two different regimes of flow velocities, pH, and light intensities in a 12 flumes experimental system for a period of 27 weeks. We used a combination of geochemical proxies, physiological and micro-probe measurements to assess how these treatments affected the conditions in the DBL and the response of organisms to OA. Overall, low flow velocity did not ameliorate the negative effect of low pH and therefore did not provide a refugia from OA. Flow velocity had species-specific effects with positive effects on calcification for two species. pH in the calcifying fluid (pH_cf_) was reduced by low flow in both corals at low light only. pH_cf_ was significantly impacted by pH in the DBL for the two species capable of significantly modifying pH in the DBL. The dissolved inorganic carbon in the calcifying fluid (DIC_cf_) was highest under low pH for the corals and low flow for the coralline, while the saturation state in the calcifying fluid and its proxy (FWHM) were generally not affected by the treatments. This study therefore demonstrates that the effects of OA will manifest most severely in a combination of lower light and lower flow habitats for sub-tropical coralline algae. These effects will also be greatest in lower flow habitats for some corals. Together with existing literature, these findings reinforce that the effects of OA are highly context dependent, and will differ greatly between habitats, and depending on species composition.

## Introduction

Ocean acidification (OA) is a major threat to many marine calcifying species, acting to reduce the calcification of ecologically important species such as reef-forming corals and coralline algae^1^. However, there is evidence that responses to OA are context-dependent, with interactions across species and the environment mediating the strength of responses^2,3^. One potentially important and still poorly studied controller of the direction and magnitude of responses is the seawater velocity that resident organisms are exposed to^4,5^. This is expected to be most important at the discrete boundary layer between marine organisms and ambient seawater, where flow is reduced to the point such that the movement of dissolved substances between the bulk seawater and the surface of the organism is dominated by molecular diffusion^6^. This layer, known as the diffusion/diffusive boundary layer (DBL), increases in thickness as seawater velocity decreases^7,8^.

There are three main reasons via which mass transfer limitation in the DBL could influence the response of calcifying organisms to OA: (1) by changing the pH environment at their surface^9,10^, (2) by limiting the transfer of essential nutrients and dissolved inorganic carbon^11,12^, (3) or by causing the build-up of waste substances, such as protons during calcification^13^. The concentration gradient within the DBL is directly related to its thickness and the metabolic activity of the organism^5,14^. Therefore, when water velocity decreases, it increases the thickness of the DBL which favours the increase of pH at the surface of photosynthetic organisms during the day, and decreases in pH at night due to respiration^10,15^. There has been significant discussion of whether such diurnal changes in DBL pH may ameliorate the impacts of OA, whereby pH during the day and mean pH encountered by the organism are increased relative to the overlying seawater^16^.

Conversely, reduced dissolved inorganic carbon or nutrients within thicker DBLs could have negative ramifications under OA. In many temperate systems, reduced nutrient concentrations at the surface of the organism might not have great impacts, but in already nutrient-poor systems such as coral reefs, these effects could have large impacts on organism metabolism. These would act additively with the impacts of OA. Reduced export of protons from the calcifying fluid to the external seawater is another possible negative ramification of thicker DBLs that could alter responses to OA, following the proton flux hypothesis^13^. This would manifest by reducing pH in the calcifying fluid (pH_cf_), with the effects expected to be most pronounced under both OA and slow flow.

Only two studies have examined the interactive effects of water velocity and OA, finding contrasting effects. Cornwall *et al*.^5^ found that the effects of OA were ameliorated for a temperate articulate coralline alga under slow flow (interactive effects). Daily increases in pH within the DBL were greater under slow flow and OA than nightly decreases in pH, thereby increasing mean pH at the surface, corresponding to faster calcification rates. Conversely, Comeau *et al*.^4^ found that a coral reef community comprised of corals, crustose coralline algae (CCA) and carbonate sediments had lower calcification under both reduced pH and under slower flow (additive effects). Three logical explanations exist for the contrasting results due to: (1) differences between organism physiology; (2) environmental conditions; or (3) the inclusion of sediment in Comeau *et al*.’s study. Coral reef communities are more likely to display mass-transfer limitation than temperate macroalgae^17^, therefore any positive effects of pH modification in the DBL may be overwhelmed by the negative impacts of nutrient limitation during Comeau *et al*.^4^. Tropical corals (and even CCA) might therefore be expected to respond differently from temperate articulate coralline algae. This could be due to the fact that the capacity of corals to modify pH at their surface might be much less than that of the coralline algae, or because their export of protons from their calcifying site might be differentially impacted by flow. Unfortunately, neither Cornwall *et al*.^5^ nor Comeau *et al*.^4^ measured pH_cf_, nor did Comeau *et al*.^4^ measure pH in the DBL of the different species in their communities.

Here we attempt to gain a greater understanding of how seawater velocity will interact with OA for key calcifying species, corals and CCA, by testing two competing questions, based on the results of seemingly contradictory studies^4,5^. Will slow flow provide a refugia from OA for calcifying species^16^, or will faster flow reduce the impacts of OA? We tease out the physiological mechanisms responsible for both past results by growing corals and CCA under an interactive combination of slow/fast flow and low/ambient pH over a longer duration than used previously (27 weeks), and measure pH both at the surface of all organisms within the DBL, and pH within their calcifying fluids. We do this by using both taxa (corals and CCA) and by also crossing the already complex design with two irradiance regimes, with both high and low light for each taxon. While light is known to influence calcification and photosynthesis rates of both taxa^18,19^, and can be particularly important in mediating the response of macroalgae to OA^20^, these regimes also allow us to have one condition where pH modification in the DBL is potentially high and another where it is low (i.e. high and low light respectively). We conduct these experiments under conditions where nutrient concentrations are similar to those in coral reef systems (low concentrations), to represent the natural conditions where the organisms were collected and to test whether mass transfer limitation could be responsible for past results^21^. We hypothesize (1) that if slow flow acts ubiquitously as a refugia, then day time pH in the DBL will be directly correlated to calcification rates and favourable calcifying fluid chemistry across all treatments, (2) that if the export of protons limits calcification under slow flow, then pH_cf_ will be lower under slow flow for all taxa, particularly when combined with OA, and (3) if neither hypothesis is correct then mass-transfer limitation is responsible for the interactive effects.

## Materials and Methods

### Organism collection and preparation

The experiment was carried out from February 15^th^ until August 25^th^ 2017 in the University of Western Australia’s Indian Ocean Marine Research Centre at Watermans Bay. Two weeks prior to the start of the experiment organisms were collected from Salmon Bay, Rottnest Island, WA, Australia (32°01′16.44″S, 115°31′16.60″E), which is located ~15 km offshore the marine laboratory (see Ross *et al*.^22^ for the site description). A total of 48 branches (~5 cm tall) of the coral *Acropora yongei* were hand-collected and 48 whole colonies (~5 cm in diameter) of the coral *Plesiastrea versipora* were chiselled out from the reef at ~1–2 m depth. *Acropora* branches were collected ~5 m apart to maximise genetic diversity. Additionally, 48 whole rhodoliths of the coralline alga (CCA) *Sporolithon durum* (~5–8 cm) were hand picked at ~1–2 m depth. Only *S. durum* with limited or no exposed skeleton were selected. Back at the laboratory, the corals were glued to plastic supports using epoxy glue and tags were attached to *S. durum* using nylon lines. Organisms were acclimated to the laboratory lighting, temperature, and flow conditions (similar to the one experienced *in situ* and then used during the experiment) in a flow trough system during two weeks under ambient pH (pH_T_ ~ 8.1). At the end of the acclimation period, skeletons of the organisms were stained by placing the samples for 24 hours in a bath of seawater enriched with calcein at 50 mg l^−1^ with pH adjusted to 8.1 by addition of NaOH. The stain line was used during the geochemical analyses as a visual indicator to select portion of the skeleton grown under the experimental conditions (i.e. section above the stain line).

### Experimental set-up

Twelve custom-made flumes were used to maintain the organisms under controlled conditions of flow, temperature, light and pH. The 90 L flumes consisted of a 1.5 m × 0.2 m × 0.2 m working section. Seawater was circulated at the upstream side of the flume through a 0.3 m long transition chamber mounted with flow straighteners made of stacked PVC pipes (diameter of 1.5 cm) covered by a shade cloth to obtain a unidirectional semi-laminar flow. The return section was made of 0.1 m diameter PVC pipes. The organisms were maintained at two flow velocities of 0.025 m s^−1^ (Slow Flow) and 0.08 m s^−1^ (Fast Flow) that were obtained using two types of underwater pumps (D1 and D3 – DC wave maker pumps, Macro Aqua, China). Flow velocities were chosen to remain within ecologically relevant conditions. Flow velocity was determined at ~15 cm depth, where the organisms were placed, and adjusted for each flume using an Acoustic Doppler Velocimeter (Nortek Vectrino, Norway).

**Figure 1:**
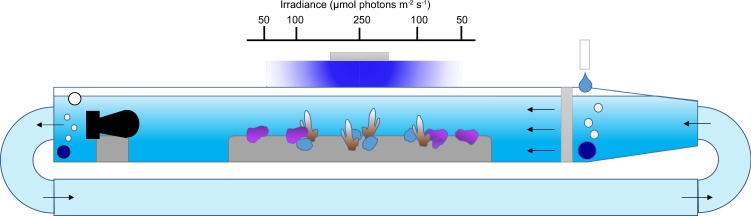
Diagram representing the experimental set-up used to maintain the corals and coralline algae for 27 weeks. The organisms were maintained under two flow velocities created by pumps placed at the end (left of the diagram) of the flumes. pH was regulated independently in each flume using a pH controller. Positions of the organisms within the flumes were used to control light levels. Organisms directly under the light received ~250 *µ*mol quanta m^−2^ s^−1^ at midday (High light treatment for corals), while organisms on the side received either ~100 *µ*mol quanta m^−2^ s^−1^ (Low light for corals, High light for CCA) at midday or ~50 *µ*mol quanta m^−2^ s^−1^ at midday (Low light for CCA).

The flume experiments were designed to test the interactive effects of two regimes of flow velocities (0.025 and 0.08 m s^−1^), pH (ambient pH: pH_T_ = 8.05 and low pH: pH_T_ = 7.65) between flumes, and light levels (Fig. 1). For clarity, in the following sections the ambient pH treatment is described as ‘pH 8.0’ and the low pH treatment as ‘pH 7.6’. Treatments of flow and pH were randomly assigned to obtain three flumes per combination of flow and pH. Light levels were controlled by the relative position of organisms within the flumes. Organisms directly under the light received ~250 *µ*mol quanta m^−2^ s^−1^ at midday (High light treatment for corals only), while organisms on the side received either ~100 *µ*mol quanta m^−2^ s^−1^ (Low light for corals, High light for CCA) at midday or ~50 *µ*mol quanta m^−2^ s^−1^ at midday (Low light for CCA only). The light levels corresponded to light intensities regularly experienced by organisms in Salmon Bay^22^. Prior experimentation determined that these light conditions were optimal for growing these species in the laboratory^23,24^. Four corals per species were randomly placed under 250 or 100 *µ*mol quanta m^−2^ s^−1^ (n = 2 per light treatments for each flume). Four coralline algae were randomly placed under 100 or 50 *µ*mol quanta m^−2^ s^−1^ (n = 2 per light treatments for each flume). With this design, 6 organisms of each species were exposed to each combination of treatments.

Temperature was maintained constant at ~20.5 °C, which is the mean *in situ* temperature at the collection site^25^, during the experiment. Light was provided by 150 W LED (Malibu LED, Ledzeal) that followed a diel cycle. Light was gradually ramped-up in the morning, commencing from 6:00 h until 10:00 am to reach maximum intensity, remained at maximum intensity for four hours, and then ramped down until total darkness at 18:00 h. pH was manipulated in the flumes using pH-controllers (AquaController, Neptune systems, USA) that control the bubbling of pure CO_2_. Ambient air was continuously bubbled in each flume to maintain the O_2_ and ambient pH constant. The flumes worked as a flow through system with sand-filtered seawater (porosity ~25 *µ*m) delivered continuously at ~0.5 L min^−1^.

### Carbonate chemistry measurement and calculations

Seawater pH and temperature were measured every ~1–2 d in each flume using a pH meter calibrated every 2 d on the total scale using Tris/HCl buffers made following^26^. Total alkalinity (*A*_T_) was measured weekly in all the flumes using an open cell potentiometric method (Mettler Toledo, T50). *A*_T_ was calculated using a modified Gran function and titrations of certified reference materials (CRM, batch 161) provided by A.G. Dickson lab yielded *A*_T_ values within 5 *μ*mol kg^−1^ of the certified value. *A*_T_, pH_T_, temperature, and salinity were used to calculate the carbonate chemistry parameters using the seacarb package running in R.

### Physiological measurements

Calcification was measured over the 27-week experimental duration using the buoyant weight method^27^. Net calcification was determined on each organism by converting the difference in weight between the beginning and the end of the incubation period to dry weight using an aragonite density of 2.93 g cm^−3^ (for the corals) and a calcite density (for the CCA) of 2.73 g cm^−3^. Net calcification was normalized to the surface area determined using the foil method^28^ for *P. versipora* and *S. durum,* and the relationship between skeleton weight and surface area for *A. yongei* determined by CT scanning.

Light short-term incubations were carried out after 2 months of exposure to the treatments to assess the response of photosynthesis and respiration. Incubations were conducted at this time to ensure adequate acclimation to the growing conditions. Each individual was placed into an incubation chamber filled with seawater originating from its respective flume. Flow and light were manipulated to approximate the respective conditions in the flumes. Organisms were chosen randomly for a total of 4 replicates of each species per treatment combination (96 individuals total) and controls with only flume seawater were run during each incubation. Incubations lasted ~1.5–2 h and changes in dissolved oxygen (using an A323 dissolved oxygen portable meter, Orion Star, Thermo Scientific, USA) and temperature between the beginning and end of each incubation were determined. All rates were normalized to surface-area of the organisms.

### Calcifying fluid pH_cf_ and DIC_cf_

Calcifying fluid pH (pH_cf_) for all organisms and DIC (DIC_cf_) for corals was calculated using the *δ*^11^B proxy method for pH_cf_ and the *δ*^11^B and B/Ca method for DIC_cf_ ^29^. Geochemical measurements were done on the material representing the average calcium carbonate deposited during the 27 weeks of incubation (as confirmed by calcein staining)^23,24^. The portions of the skeleton grown under the experimental conditions were sampled using cutting pliers (for *A. yongei*) or a dental drill (for *S. durum* and *P. versipora*).

All powders of selected material were processed in the clean laboratory of the Advanced Geochemical Facility for Indian Ocean Research [AGFIOR, University of Western Australia (UWA)] for dissolution and dilution to 10-ppm Ca solutions. Ten mg of each sample was placed in 6.25% NaClO for 15 mins, rinsed in MilQ water 3 times and then dried for 24 h. Samples were then dissolved in 0.51 N HNO_3_, and the boron was quantitatively separated on ion exchange columns. *δ*^11^B was measured on a multicollector inductively coupled plasma mass spectrometry (NU II). Measurements of the international carbonate standard JCp-1 yielded a mean value of 24.35 ± 0.13‰ (mean ± SE, n = 5), which was similar to the nominal value of 24.33 ± 0.11‰ (SE) reported previously^30^. Calculations of pH_cf_ based on *δ*^11^B were made using the calculations of^31^:

1 $${{\rm{pH}}}_{{\rm{cf}}}={{\rm{pK}}}_{{\rm{B}}}-\,\log [\frac{({{\rm{\delta }}}^{11}{{\rm{B}}}_{{\rm{SW}}}-{{\rm{\delta }}}^{11}{{\rm{B}}}_{{\rm{carb}}})}{({{\rm{\alpha }}}_{({\rm{B}}3-{\rm{B}}4)}{{\rm{\delta }}}^{11}{{\rm{B}}}_{{\rm{carb}}}-{{\rm{\delta }}}^{11}{{\rm{B}}}_{{\rm{SW}}}+1000\,({{\rm{\alpha }}}_{({\rm{B}}3-{\rm{B}}4)}-1))}]$$

where pK_B_ is the dissociation constant dependent on temperature and salinity, *δ*^11^B_sw_ = 39.61^32^, and α_B3_-_B4_ is the boron isotopic fractionation factor for the pH dependent equilibrium of the borate (B(OH)_4_^−^) relative to the boric acid (B(OH)_3_) species in the calcifying fluid, with a value of 1.0272^33^.

B/Ca ratios and *δ*^11^B measured on the same material was utilized to determine [CO_3_^2−^] and then [DIC] at the site of calcification [DIC]_cf_ following^29,34^. B/Ca ratios were determined on the same aliquot of the solution used for pH_cf_ estimates, and DIC_cf_ was calculated from estimates of CO_3_^2−^ using the following equations described in^29^:

2 $${[{{\rm{CO}}}_{3}^{2-}]}_{{\rm{cf}}}={{\rm{K}}}_{{\rm{D}}}{[{\rm{B}}{({\rm{OH}})}_{4}^{-}]}_{{\rm{cf}}}/{({\rm{B}}/{\rm{Ca}})}_{{{\rm{CaCO}}}_{3}}$$

Where *K*_D_ = *K*_D.0_ exp(−*k*_*KD*_[H^+^]_T_) with = 2.97 ± 0.17 × 10^−3^ (±95% CI), ${k}_{{K}_{D}}$ = 0.0202 ± 0.042. The concentration of DIC_cf_ was then calculated from estimates of pH_cf_ and [CO_3_^2−^]_cf_ using the R package seacarb.

### Raman spectroscopy

We utilized confocal Raman spectroscopy to determine sample mineralogy (aragonite versus calcite) and as a proxy of calcifying fluid saturation state (Ω). Measurements were conducted on a WITec Alpha300RA + using a 785 nm laser, 1200 mm^−1^ (~1.3 cm^−1^ spectral resolution), and a 20x objective with 0.5 numerical aperture following^35^. The wavenumber was routinely calibrated with a silicon chip (nominal peak at 520.5 cm^−1^). Topography maps were made with the TrueSurface module for skeleton samples placed on glass slides (powders for corals, and cut sections for CCA). The topography maps were then followed with an automated stage while conducting Raman measurements to ensure the optics remained in focus. For each sample, 100 spectra were collected in a square grid, 300 µm by 300 *µ*m using 1 s integrations for corals and 1 mm by 1 mm using 2 s integrations for CCA. Spectra with poor signal (<100 arbitrary intensity units or signal/noise ratio of ~10) or contaminated by cosmic rays were excluded.

Sample mineralogy was evaluated by first confirming each sample is CaCO_3_ based on the *v*_1_ peak at ~1085–1090 cm^−1^. Next, each sample was distinguished between aragonite and calcite based on the shape and position of the *v*_4_ peak between 700–720 cm^−1^, where a double peak <710 cm^-1^ is indicative of aragonite and a single peak >710 cm^−1^ is indicative of calcite. We found only aragonite in our coral samples and only high-Mg calcite in our CCA samples, confirming the mineralogy expected for each species.

The widths of the *v*_1_ peaks were used as proxy measures of calcifying fluid Ω^19^. We used the abiogenic aragonite calibration equation of^35^ to calculate Ω_a_ for the two coral species from the *v*_1_ full width at half maximum intensity (FWHM). Although there is no published abiogenic high-Mg calcite Ω calibration, we used the Mg concentration-normalized peak widths as relative indicators of Ω for CCA^36,37^. The effect of Mg on *v*_1_ FWHM was accounted for using the equations of^38^, where [Mg] is determined from *v*_1_ wavenumber, and the residual *v*_1_ FWHM is determined for qualitative calcite Ω interpretations

### Calcifying fluid Ca_2_ + cf

[Ca^2+^]_cf_ was calculated as:

3 $${[{{\rm{Ca}}}^{2+}]}_{{\rm{cf}}}={\Omega }_{Ar}\ast {K}_{sp}/{[{{\rm{CO}}}_{3}^{2-}]}_{{\rm{cf}}}$$

where ${[{{\rm{CO}}}_{3}^{2-}]}_{{\rm{cf}}}$ and Ω_*Arag cf*_ are derived from boron systematics and Raman spectroscopy, respectively.

### Micro-sensor measurements

The DBL pH was determined after 16–27 weeks of incubations using a Unisense microprofiling system (Unisense A/S, Denmark). Measurements were made directly in the flumes on the organisms maintained under their respective conditions of light, pH, and flow. pH_T_ of the flumes bulk seawater was measured following the method described above before each profile determinations. pH in the DBL was measured with a pH-50 microelectrode with a 40–60 *μ*m tip diameter and an external reference electrode (Radiometer analytical). The pH microelectrode was calibrated using NBS buffers every day, and standardised to the total scale based on Tris mV prior to any further measurements.

A Unisense manual micro-manipulator and a hand-held magnifying glass were used to position the sensors. For *A. yongei*, micro pH electrodes were positioned between polyps as close as possible to the surface of the organisms, while for *P. versipora* the probes were positioned inside the gap made by their large polyps. For *S. durum*, the sensors were placed as close as possible to the surface in the gap between protuberances. After a 45 min acclimation period, measurements of pH were made every 100 *μ*m above the organisms (50 *μ*m for the first 4 steps) surface up to 1800 *μ*m, with a final measurement at 2800 *μ*m. At each step, measurements were collected for 2 min. The difference in pH between the surrounding seawater and the diffusion boundary layer (DBL) were determined on at least three *P. versipora* and *S. durum* for each experimental treatment. Measurements on *A. yongei* were only done on three individuals because it was not possible to detect a consistent change in pH in the DBL. pH in the DBL in the dark was also determined on two *P. versipora*., two *S. durum* and one *A. yongei*. A 2-month period was necessary to perform all the measurements so they could be conducted roughly at the same time of day.

### Statistical analyses

Factorial ANOVA models were used to detect differences in calcification, photosynthesis, pH_cf_, DIC_cf_, Ca^2+^_cf_, Ω_cf_, and DBL pH for the corals, and calcification, photosynthesis, pH_cf_, B/Ca, Raman-derived FWHM and DBL pH for *S. durum*. pH, flow and light were fixed factors in the models, where flume of origin was also included as a random factor. The random factor was dropped from the analyses when it was not significant (p < 0.25). All data conformed to normality and homogeneity of variance. All analyses were done in R. All data will be archived in the Pangaea database.

## Results

During the 27 weeks of the experiment, carbonate chemistry was successfully maintained constant across the treatments (Table 1). pH was maintained on average at 8.04 ± 0.02 (mean ± SE, n = 384) in the pH 8.0 treatment and 7.62 ± 0.03 (mean ± SE, n = 384) in the pH 7.6 treatments, corresponding to respective pCO_2_ of 421 ± 26 and 1282 ± 83 *μ*atm. Over the course of the experiment, the nutrient concentrations were: NH_4_^+^  = 3.07 ± 2.6 μg l^−1^ (n = 8), NOx = 0.83 ± 1.42 *μ*g l^−1^, and PO_4_ = 6 ± 1.42 *μ*g l^−1^. No bleaching or mortality of corals as a function of the treatment was found, though 7 CCA experienced mortality (not linked to the treatment) and were subsequently excluded from further analysis.

**Table 1:** Mean carbonate chemistry in the flumes during the 27 weeks experiment.

Flume	Flow	pH_T_	A_T_ (*μ*mol kg^−1^)	pCO_2_ (*μ*atm)	Temperature (°C)
1	Slow	7.61 ± 0.01	2358 ± 3	1309 ± 29	20.5 ± 0.1
2	Slow	7.61 ± 0.01	2361 ± 2	1278 ± 24	20.5 ± 0.1
3	Fast	8.03 ± 0.01	2357 ± 2	427 ± 13	20.8 ± 0.1
4	Slow	7.61 ± 0.02	2360 ± 2	1350 ± 57	20.6 ± 0.2
5	Slow	8.04 ± 0.00	2361 ± 2	416 ± 5	20.6 ± 0.1
6	Slow	8.04 ± 0.00	2357 ± 3	412 ± 6	20.7 ± 0.2
7	Fast	7.63 ± 0.01	2360 ± 2	1222 ± 27	20.9 ± 0.1
8	Fast	7.61 ± 0.01	2359 ± 2	1311 ± 34	20.8 ± 0.2
9	Fast	7.63 ± 0.01	2359 ± 2	1221 ± 23	20.7 ± 0.1
10	Fast	8.03 ± 0.01	2358 ± 2	439 ± 20	20.6 ± 0.2
11	Fast	8.04 ± 0.00	2357 ± 2	410 ± 5	20.6 ± 0.2
12	Slow	8.03 ± 0.00	2356 ± 2	422 ± 6	20.5 ± 0.2

pCO_2_ was calculated using measured pH_T_, total alkalinity (A_T_), temperature and a salinity of 36.3 (SE < 0.1). All values are mean ± SE (n = 64).

### Calcification

**Figure 2:**
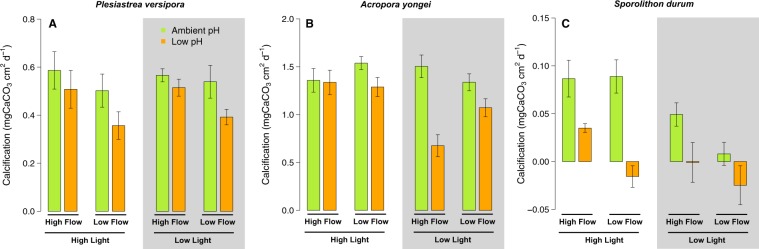
Effects of pH, flow and light on the surface-area normalized net calcifcation rates of the corals *Plesiastrea versipora* and *Acropora yongei* and the coralline alga *Sporolithon durum*. Calcification was measured on organisms grown in flumes for 27 weeks. pH was maintained at ambient pH (green bars, pH = 8.1) and low pH (orange bars, pH = 7.65). Seawater velocity was adjusted to high flow (8 cm s^−1^) and low flow (2.5 cm s^−1^). Light levels were manipulated within the flumes to high light (250 *μ*mol photon m^−2^ s^−1^ for corals and 100 *μ*mol photon m^−2^ s^−1^ for the CCA) and low light (100 *μ*mol photon m^−2^ s^−1^ for corals and 50 *μ*mol photon m^−2^ s^−1^ for the CCA) levels. Values displayed are mean ± SE (n = 6).

For *P. versipora*, the highest calcification was found in the pH 8.0 - Fast Flow conditions under High and Low Light, while calcification was the slowest in the pH 7.6, High Light, Slow Flow treatment (Fig. 2A). Calcification was significantly affected by pH (p = 0.011, Table S1), but there was no significant effect of Light (p = 0.675). Flow significantly affected calcification (p = 0.034) that was on average higher at Fast Flow than Slow Flow. There was no significant interactive effect between the tested parameters.

For *A. yongei*, the highest mean net calcification was measured in the pH 8.0 – High Light – Slow Flow treatment and the lowest in the pH 7.6 – Low Light –Fast Flow (Fig. 2B). Net calcification was affected by pH (p < 0.001, Table S1), Light (p = 0.003), and their interaction (p = 0.007) with calcification on average higher at pH 8.0 and High Light compare to pH 7.6 and Low Light. Flow did not impact calcification (p = 0.293), but there was a significant interaction between pH, Light and Flow because the negative effects of flow were larger at Low Light and pH 7.6 (p < 0.016).

Calcification of the CCA *S. durum* was fastest in the High Light treatment at pH 8.0 (for the Fast and Slow Flow) and negative in the two pH 7.6 - Slow Flow treatments (Fig. 2C). CCA calcification was significantly affected by pH (p < 0.001), Light (p < 0.001), and Flow (p = 0.019). However, there were no statistically significant interactive effects.

### Calcifying fluid pH

**Figure 3:**
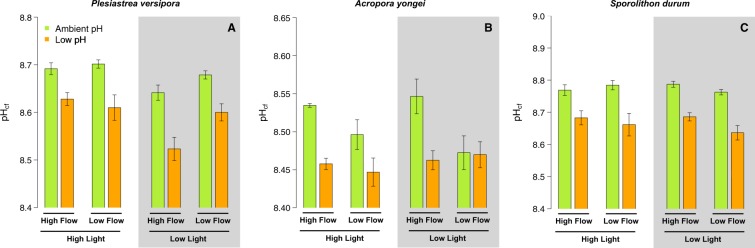
Estimates of pH in the calcifying fluid (pH_cf_) derived from *δ*^11^B for the corals *Plesiastrea versipora* and *Acropora yongei* and the coralline alga *Sporolithon durum*. pH_cf_ was determined at the end of the 27 week incubation period on organisms maintained at ambient pH (green bars, pH = 8.1) and low pH (orange bars, pH = 7.65). Seawater velocity was adjusted to high flow (8 cm s^−1^) and low flow (2.5 cm s^−1^). Light levels were manipulated within the flumes to high light (250 *μ*mol photon m^−2^ s^−1^ for corals and 100 *μ*mol photon m^−2^ s^−1^ for the CCA) and low light (100 *μ*mol photon m^−2^ s^−1^ for corals and 50 *μ*mol photon m^−2^ s^−1^ for the CCA) levels. Values displayed are mean ± SE (n = 6).

For *Plesiastrea,* the lowest pH_cf_ was found in the pH 7.6 – Low Light – Fast Flow treatment and the highest in the pH 8.0 under High Light at both flows. As a result, there was an effect of pH (p < 0.001, Table S2), Light (p = 0.001), and the interaction between Light and Flow (p ~0.048) (Fig. 3A).

For *A. yongei*, estimates of pH_cf_ were the highest in the pH 8.0 - Fast Flow treatments under both Light conditions (Fig. 3B). There was a significant effect of pH (p < 0.001, Table S2) and Flow (p = 0.034) as well as an interactive effect between Flow and pH (p = 0.017) with pH_cf_ being reduced by slow flow rates under seawater pH 8.0.

pH_cf_ of the coralline *S. durum* ranged from 8.64 (pH 7.6 – Low Light – Slow Flow) to 8.78 (pH 8.0 – High Light – Slow Flow and pH 8.0 – Low Light- Fast Flow). pH_cf_ was only affected by pH (p < 0.001, Table S2) with the lowest values recorded in all the pH 7.6 treatments (Fig. 3C).

### Calcifying fluid DIC

**Figure 4:**
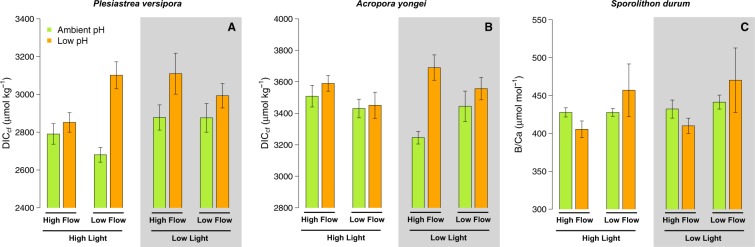
Estimates of DIC in the calcifying fluid (DIC_cf_) derived from *δ*^11^B and B/Ca for the corals *Plesiastrea versipora* and *Acropora yongei*. B/Ca is used here as a proxy of DIC_cf_ for the coralline alga *Sporolithon durum*. Note that an increase in B/Ca indicates a decrease in DIC_cf_. Values displayed are mean ± SE (n = 6).

For *P. versipora*, DIC_cf_ was affected by pH (p = 0.001, Table S3) and Light (p = 0.044). The interaction between pH, Light and Flow (p = 0.044) was also significant because the effects of Flow and pH were reversed at Low Light (Fig. 4A). Estimates of DIC_cf_ for *A. yongei* were only affected by pH (p = 0.003, Table S3) with DIC_cf_ more elevated on average in the pH 7.6 treatments (Fig. 4B).

B/Ca of *S. durum*, which is expected to be indicative of DIC_cf_, was the highest under the pH 7.6 – Slow Flow conditions and was similar in all the pH 8.0 treatments (Fig. 4C). Flow significantly affected B/Ca (p = 0.039, Table S3) and there was a trend towards a significant interactive effect of Flow and pH (p = 0.063) because B/Ca was more elevated at pH 7.6 only under Slow Flow.

### Ω_cf_ and residual FWHM

**Figure 5:**
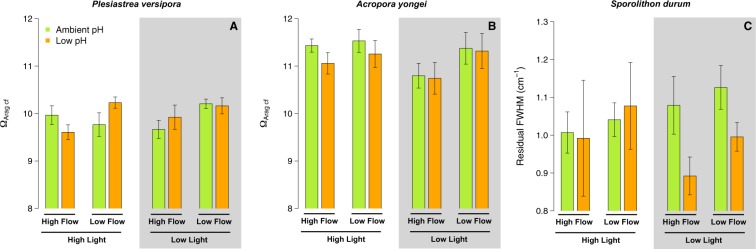
Estimates of the aragonite saturation state in the calcifying fluid (Ω_arag cf_) for the corals *Plesiastrea versipora* and *Acropora yongei*. Ω_arag cf_ was estimated using Raman microscopy. Residual FWHM is directly used as a proxy of Ω_calc cf_ for the coralline alga *Sporolithon durum* because no empirical relationship between residual FWHM and Ω_calc_ exist. Values displayed are mean ± SE (n = 6).

For *P. versipora*, Ω_cf_ was affected by Flow (p = 0.034, Table S4) because Ω_cf_ was on average more elevated under low flow. However, there were no effects of pH and Light on *P. versipora* Ω_cf_ (Fig. 5A, Table S4). The Ω_cf_ of *A. yongei* was not significantly affected by any treatment (Fig. 5B).

The treatments did not significantly affect the residual FWHM (indicator of the high-Mg calcite saturation state in the calcifying fluid) of *S. durum* (Fig. 5C, Table S4).

### Calcifying fluid Ca^2+^

**Figure 6:**
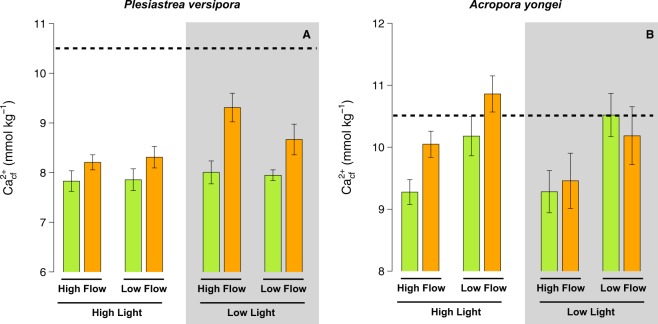
Estimates of the calcium concentration in the calcifying fluid (Ca^2+^_cf_) for the corals *Plesiastrea versipora* and *Acropora yongei*. Ca^2+^_cf_ was calculated from estimates of Ω_arag cf_ and estimates of DIC_cf_. Values displayed are mean ± SE (n = 6). The horizontal dashed line shows the approximate [Ca] in seawater during the experiment. Values displayed are mean ± SE (n = 6).

Ca^2+^_cf_ in *P. versipora* was significantly affected by pH (p < 0.001, Table S5) and Light (p = 0.014). Ca^2+^_cf_ was more elevated under low pH and low light (Fig. 6A). For *A. youngei*, Ca^2+^_cf_ was only affected by flow, with the highest values measured under low flow (Fig. 6B).

### Photosynthetic rates

**Figure 7:**
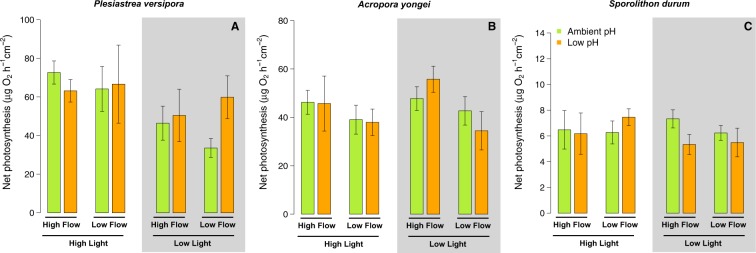
Net Photosynthesis rates determined on the corals *P. versipora* and *A. yongei* and the coralline alga *S. durum*. Net photosynthesis was determined during incubations performed after 2 months in the experimental treatments. Values displayed are mean ± SE (n = 6).

Net photosynthetic rates of *P. versipora* were only affected by Light (p = 0.043), with lower rates recorded in the Low Light treatment. For *A. yongei*, net photosynthetic rates were only significantly affected by Flow (p = 0.042) because of lower rates under Low Flow (Fig. 7B, Table S6). Net photosynthetic rates of *S. durum* were not affected by the treatments (Fig. 7C, Table S6).

### Metabolic alteration of pH in the DBL

**Figure 8:**
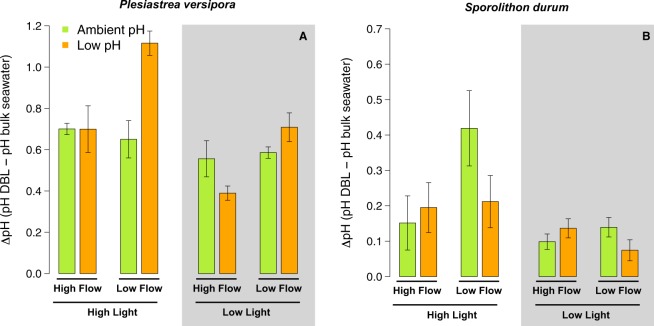
Maximal differences in pH between the diffusive boundary layer (DBL) and the bulk seawater in the light for the corals *Plesiastrea versipora* and the coralline alga *Sporolithon durum*. Values displayed are mean ± SE (n = 3).

Delta pH (pH values recorded near the organism surface with micro-sensors minus mainstream seawater pH) in the DBL in the light was not successfully measured on *A. yongei* because the changes in pH were too small. The only profile completed in the dark showed a strong decrease of ΔpH in the DBL of 0.7 unit. For, *P. versipora*, there was a significant effect of pH (p = 0.050, Table S6), Flow (p = 0.002), and their interaction (p = 0.002) on ΔpH. This interactive effect was due to greater ΔpH in the low pH treatments under low flow (Fig. 8A). As a consequence pH in the DBL was similar between pH treatments under low flow (Fig. S1). Light (p < 0.001) and the interaction between Light and pH (p = 0.021) also affected ΔpH. This was caused by higher ΔpH under high light, particularly in the low pH treatment. The two measurements of ΔpH in the dark in the pH 8.0 – Fast Flow treatments showed a decrease in pH in the DBL of ~0.6 unit.

For *S. durum*, only Light had a significant effect on ΔpH in the DBL (p = 0.006), with higher ΔpH in the high light treatments. There was also a trend toward an interaction between pH and Flow (p = 0.061) because ΔpH was higher at pH 8.0 in the Slow Flow conditions. In the dark, ΔpH in the DBL was 0.07 and 0.05 for the two test measurements made on *S. durum* from the pH 8.0 – Fast Flow treatments.

## Discussion

Understanding the role played by different physical parameters is necessary to explain the large range of responses to OA measured in past studies, and to accurately predict how OA will manifest across habitats with differing environmental conditions. We demonstrate here that flow, light, and pH and their interactions are critical factors impacting organisms’ physiology, internal chemistry, and conditions in the DBL. As a result of these complex interactions, calcification responded differently to the treatments in all the tested organisms because of morphological and physiological differences. In contrast to our initial hypotheses (1) low flow conditions did not alleviate the negative effects of OA on calcification and (2) the export of protons is likely not the main driver of calcification in all species, because there was not a consistent effect of flow on pH_cf_. In contrast, our results suggest that mass-transfer limitation of nutrients or night-time dissolution play an important role in the control of net calcification rates.

### Low flow does not provide a general refugia from OA

In the present experiment, low flow did not alleviate the effect of OA for the three species. These results are in contrast to Cornwall *et al*.^5^, who showed that low flow conditions ameliorate the negative effect of OA on calcification of the temperate articulate coralline alga *Arthrocardia corymbosa*. Here, the coralline alga exhibited negative rates of calcification under both irradiances under low flow and low pH conditions. The discrepancy between the two studies can likely be explained by two non-exclusive hypotheses. First, the present study was performed on sub-tropical algae that were grown in low nutrient concentrations similar to undisturbed coral reefs^39^, while Cornwall *et al*.^5^ used temperate algae grown under higher nutrient concentrations. It is therefore likely that under low flow *S. durum* were nutrient limited, which in turn limited their calcification rates. Nutrient uptake in oligotrophic waters is indeed dependent on flow^21^ and can have large impacts on calcification rates. Second, it is possible that the increase of pH in the DBL measured here was not sufficient to limit the effect of OA on calcification. Despite a linear relationship between pH in the DBL and calcification in *S. durum* (Fig. S2), the increase in pH in the DBL was not sufficient to ameliorate the effect of OA. Here, the relatively low photosynthetic activity of coralline algae in all the treatments and the flow velocities used (minimum of 2.5 cm s^−1^) can explain the limited increase of pH in the DBL under OA. Furthermore, it is possible that the increase in pH during the day was not sufficient to counteract the lower pH at night in the DBL, and therefore that mean DBL pH was not elevated on average over a 24 hours cycle under slow flow. Unfortunately, pH in the DBL at night was not measured because of logistical and experimental constraints.

Our results are partially in agreement with Comeau *et al*.^4^ who found that calcification of coral communities is enhanced by faster flow under ambient and OA conditions. Here, this is true for one of the two coral species, as the effects of OA on calcification of *P. versipora* were ameliorated under high flow. However, this was not true for *A. yongei*. These species-specific responses were likely driven by different morphologies (branching vs mounding) and different physiologies. The mounding physiology of *P. versipora*, with deep polyps, likely created areas with alternatively low and thick DBL. However, enhanced pH in the DBL during the day was not associated with increased calcification in *P. versipora*. Calcification was the lowest in the low flow and low pH treatments where the largest DBL ΔpH was measured. This result indicates a control of calcification by either bulk seawater pH (lower calcification recorded in the low pH treatment), nutrient concentrations (lower nutrient available at low flow) and/or low pH in the DBL at night. The few measurements of pH in the DBL in the dark for *P. versipora* showed a negative delta pH of similar magnitude to the light ΔpH, which could indicate that the positive effect of higher pH during the day is balanced by the negative effects of lower pH at night under slow flow. However, further studies will be necessary to confirm this observation.

### The export of protons is not the main driver of calcification in all species

Our results demonstrate that the chemistry at the site of calcification is strongly impacted by the physical and chemical conditions in which the organisms are living. The relationship between pH_cf_ and seawater pH is species-specific in corals and CCA^24,40,41^. This trend was repeated here with the three tested taxa exhibiting different pH_cf_. For example, mean pH_cf_ in the ambient seawater pH treatments was 8.68, 8.51, and 8.78 for *P. versipora*, *A. yongei*, and *S. durum* respectively. These values are within the range of what has been reported previously using a variety of techniques such as micro-electrodes^42^, pH-sensitive dye^43^, and boron isotope proxies^44^. In addition, the present study also demonstrates that pH_cf_, and the general chemistry at the site of calcification (DIC_cf_, Ca^2+^_cf_, Ω_cf_), is also modulated by flow and/or light depending on the species. This has important repercussions, as it demonstrates that using pH_cf_ or DIC_cf_ as indicators of the seawater chemistry^45^ can be confounded by other physical parameters. This result also suggests that seasonal variation in pH_cf_ and DIC_cf_^29^ could be partly driven by seasonal variations in both light and flow, which should now be measured in the future *in situ* proxy research to improve accuracy of any reconstructions of the physical or chemical environment.

Generally, seawater pH was the main driver of pH_cf_ in the three tested species, while the effects of flow and light were species-specific and subtler. Light had the strongest effects on *P. versipora*, with pH_cf_ being lower within low light treatments. This could have been driven by generally lower DBL ΔpH under low light for *P. versipora*. The linear relationship between DBL ΔpH and pH_cf_ for *P. versipora* and *S. durum* (Fig. S3) suggests that the elevation of pH at the surface of the organisms can slightly modify pH_cf_ and favour the export of protons from the site of calcification by reducing the proton gradient between the calcifying fluid and the mainstream seawater. This indicates that pH near the surface of the organism could be more important than bulk seawater pH in influencing pH_cf_, particularly for mounding species. However, this is not found in all the corals, as it was not possible to link pH_cf_ and DBL ΔpH in *A. yongei*, because DBL ΔpH was nearly impossible to measure (i.e. close to 0) here, something which has been recently repeated with another *Acropora* species^46^. We also did not find any direct relationships between calcification and pH_cf_ for the two corals (Fig. S4). This is in agreement with our previous work that showed that pH_cf_ does not always drive calcification^23,37,47^. For *S. durum*, there was also no linear relationship between pH_cf_ and calcification but calcification and pH_cf_ were the lowest in the low pH treatments.

### Chemistry in the calcifying fluid

[Ca^2+^]_cf_ of *P. versipora* increased at lower seawater pH, though it remained lower than seawater [Ca^2+^], as found previously in *Pocillopora damicornis*^48^. This could be one of the mechanisms used by this species to maintain elevated Ω_cf_ and calcification under low pH. *A. youngei* [Ca^2+^]_cf_ also showed similar patterns to that observed previously in this species, as [Ca^2+^]_cf_ was not affected by seawater pH^48^. In contrast, the higher [Ca^2+^]_cf_ of *A. youngei* under low flow conditions were not correlated with higher calcification rates. Together with previous observations^42^, this collectively demonstrates that [Ca^2+^]_cf_ is both driven by environmental parameters and is highly species-specific. Nevertheless, [Ca^2+^]_cf_ and [CO_3_^2−^]_cf_ were linearly correlated in both species (Fig. S5), demonstrating that increasing [Ca^2+^]_cf_ can be used by both species as a compensatory mechanism in response to declining [CO_3_^2−^]_cf_.

There were strong species-specific effects of the experimental conditions on DIC_cf_. The few studies that have investigated DIC_cf_ have assumed that DIC_cf_ was driven by photosynthesis, seawater pH, or seawater DIC^29,47,49,50^. Here we also show that flow and light can have complex effects on DIC_cf_ and that these effects are pH dependent. The relationship between B/Ca and the employed treatments for the coralline alga was also complex. Under high flow, B/Ca was lower (DIC_cf_ higher) at low seawater pH, which is similar to the effects of seawater pH on corals^51^. However, here the opposite was found for *S. durum* at low flow, demonstrating that seawater carbonate chemistry is not the only driver of CCA DIC_cf_. This lower B/Ca could be the result of the very low consumption of DIC by calcification in the low flow, low pH treatments where the lowest rates of calcification were measured.

The overall lack of an effect of the treatment on Ω_arag cf_ or FWHM while calcification varied between treatments is indicative of three processes. First, Ω_arag cf_ and FWHM represents the chemical condition in the calcifying fluid when the precipitation of calcium carbonate occurred. Here, our results show that corals and CCA need to reach a certain species-specific threshold Ω_arag cf_ (or FWHM) to initiate the precipitation process (i.e., Ω_arag cf_ ~ 11 for *A. yonge*i and ~10 for *P. versipora*, FWHM ~ 1.0 for *S. durum*), regardless of the external conditions of flow, light and pH. Second, Ω_arag cf_ (or FWHM) does not provide information on the bulk rate of calcification (i.e. the instantaneous precipitation rate integrated over time and surface area). Finally, the discrepancy between Ω_arag cf_ (or FWHM) and calcification could be the sign that dissolution in some treatments played a role, as this would decrease calcification without affecting the chemistry at the site of calcification during calcification.

## Conclusion

The present study shows that light, flow, and pH have complex species-specific effects on corals and coralline algae. Slow flow conditions did not provide refugia from ocean acidification, and in contrast it had no effect or negative effects on calcification. Nor did elevated seawater velocity show clear evidence of increased proton export. Here we clearly demonstrate the role of flow is context and species-specific. This highlights the necessity of assessing hypotheses regarding how climate change will manifest across multiple species under a variety of environmental conditions, while at the same time evaluating the physiological effects of these parameters. Further, the strong effects of irradiance and flow on the carbonate chemistry within the calcifying fluid confirm the difficulties associated with using skeletal proxies to estimate environmental and physiological conditions^23,45,51^. Therefore, caution must be applied to assuming constant offsets of these parameters from seawater carbonate chemistry across different sites, even within the same species. Most importantly however, we demonstrate here that the effects of ocean acidification will manifest differently between habitats with differences in light and seawater velocity. These differences are complex, difficult to predict based on existing hypotheses regarding the impacts of seawater velocity, and are species-specific. This indicates the need for more targeted research that further assesses the impacts of seawater velocity; based on our findings further work should couple similar research with assessment of dissolution and/or manipulations of nutrient concentrations.

## Supplementary information

Supplementary Figures & Tables
