# Supplementary material for: Flow-driven micro-scale pH variability affects the physiology of corals and coralline algae under ocean acidification
Source: Sci Rep. 2019 Sep 6;9:12829. doi: 10.1038/s41598-019-49044-w (PMC6731248; doi:10.1038/s41598-019-49044-w)
Supplement: Supplementary file 1 — Supplementary Figures & Tables [file 41598_2019_49044_MOESM1_ESM.pdf]

Flow-driven micro-scale pH variability affects the physiology of corals and coralline algae under ocean acidification

Comeau S.[1,2,3,†,*], Cornwall C.E. [1,2,4,†], Pupier C.A. [1,5,6], DeCarlo T.M. [1,2], Alessi C. [1], Trehern, R.[1], McCulloch M. T.[1,2]

[1]The University of Western Australia, Oceans Graduate School and Oceans Institute, 35 Stirling Highway, Crawley 6009, Western Australia, Australia

[2]ARC Centre of Excellence for Coral Reef Studies, 35 Stirling Highway, Crawley 6009, Western Australia, Australia

[3]Sorbonne Université, CNRS-INSU, Laboratoire d'Océanographie de Villefranche, 181 chemin du Lazaret, F–06230 Villefranche-sur-mer, France

[4]School of Biological Sciences, Victoria University of Wellington, Wellington, New Zealand

[5]Centre Scientifique de Monaco, 8 Quai Antoine Ier, MC-98000 Monaco, Monaco

[6]Sorbonne Université, Collège doctoral, F-75005 Paris, France

[†]SC and CEC contributed equally

[*]Corresponding author: comeau@obs-vlfr

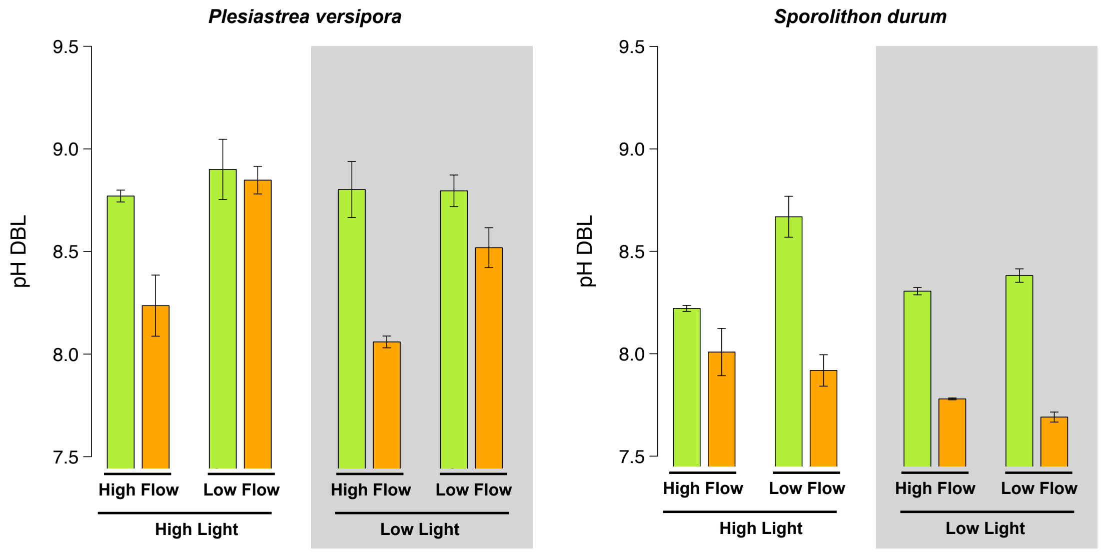

Fig. S1. Effects of pH, flow and light on the pH in the diffusive boundary layer (pH DBL) of the coral *Plesiastrea versipora* and the coralline alga *Sporolithon durum*. pH was maintained at ambient pH (green bars, pH =8.1) and low pH (orange bars, pH = 7.65). Seawater velocity was adjusted to high flow (8 cm s^{-1}) and low flow (2.5 cm s^{-1}). Light levels were manipulated within the flumes to high light (250 μmol photon m^{-2} s^{-1} for corals and 100 μmol photon m^{-2} s^{-1} for the CCA) and low light (100 μmol photon m^{-2} s^{-1} for corals and 50 μmol photon m^{-2} s^{-1} for the CCA) levels.

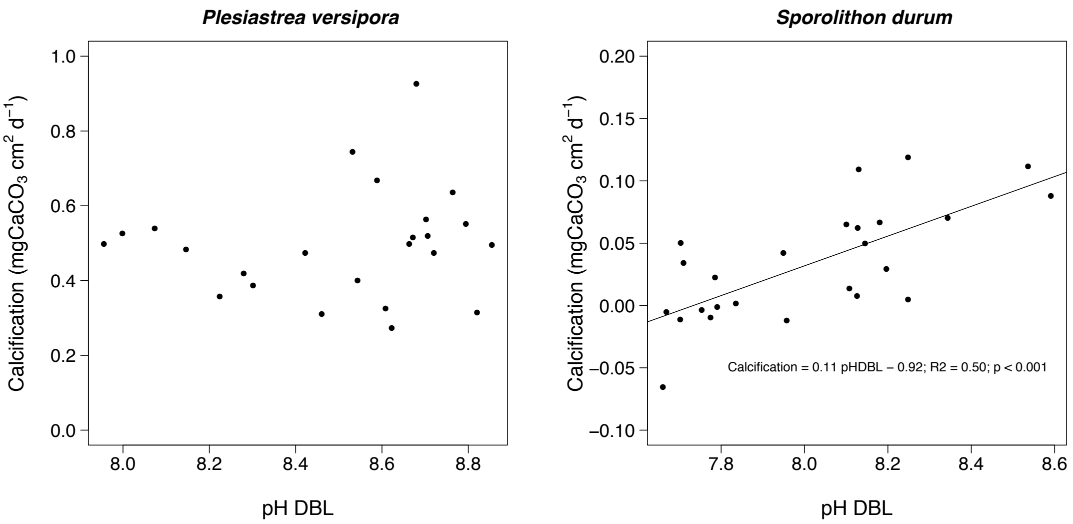

Fig S2. Plots showing the relationships between calcification and pH DBL for the corals *Plesiastrea versipora* and the coralline *Sporolithon durum.* Modifications of pH DBL were not detected in *Acropora yongei*.

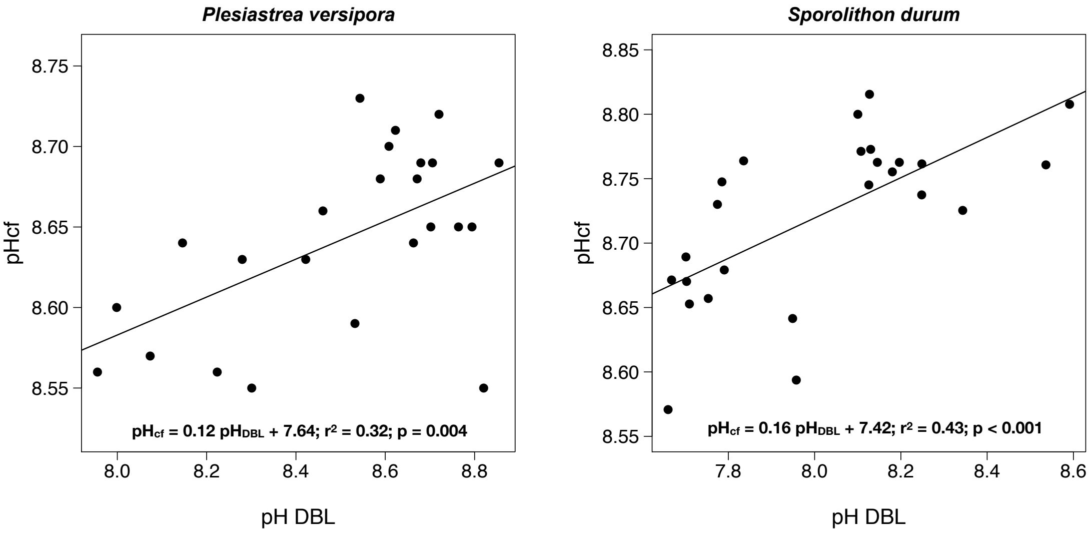

Fig. S3. Relationship between pH_{cf} and pH on the diffusive boundary layer in the coral *Plesiastrea versipora* and the coralline alga *Sporolithon durum*.

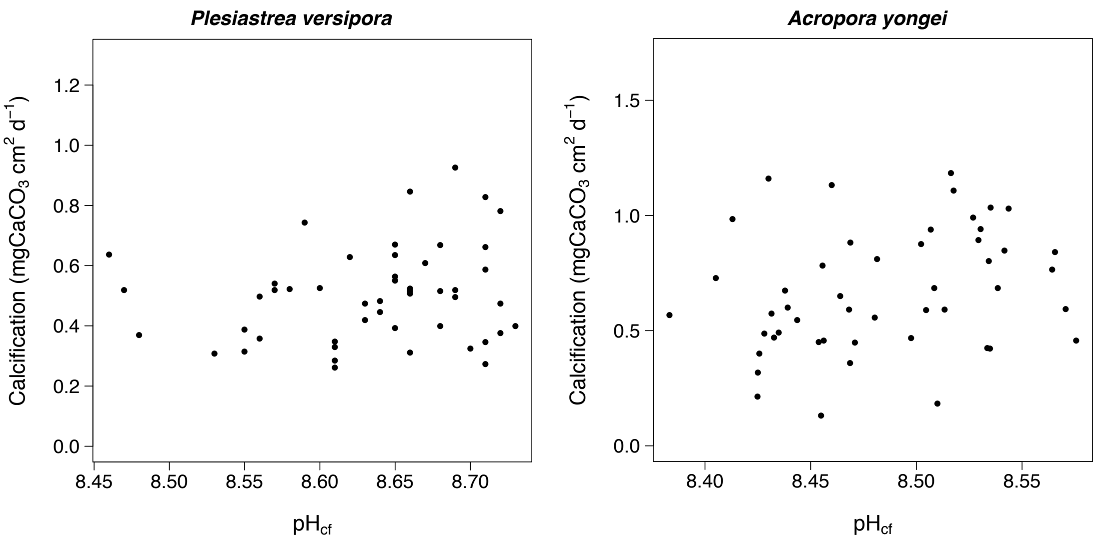

Fig. S4. Plots showing the lack of a relationship between calcification and pH_{cf} for the corals *Plesiastrea versipora* and *Acropora yongei*.

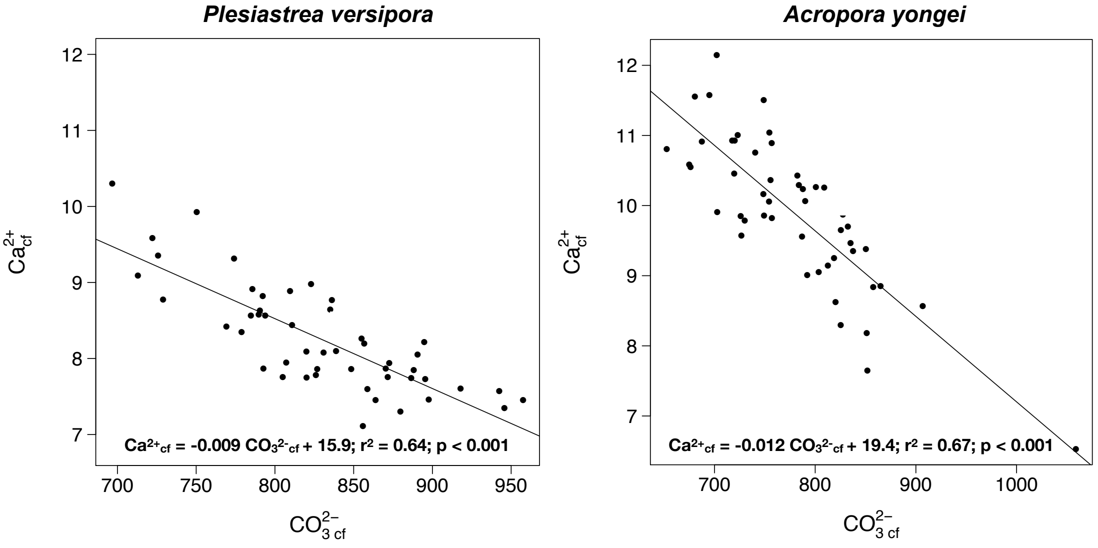

Fig. S5. Plots showing the relationships between $Ca^{2+}{}_{cf}$ (mmol kg^{-1}) and $CO_3{}^{2-}{}_{cf}$ (micromol kg^{-1}) across treatments for the corals *Plesiastrea versipora* and *Acropora yongei*.

Table S1. ANOVA table showing the effect of treatments on calcification of the corals *Plesiastrea versipora* and *Acropora yongei*, and the coralline alga *Sporolithon durum*.

Species	Effect	df	MS	F	p
P. versipora	pH	1	0.15	7.04	**0.011**
	Light	1	0.00	0.18	0.675
	Flow	1	0.10	4.78	**0.034**
	pH x Light	1	0.00	0.04	0.853
	pH x Flow	1	0.02	1.06	0.309
	Light x Flow	1	0.01	0.29	0.594
	pH x Light x Flow	1	0.00	0.03	0.862
	Residuals	43	0.02		
A. yongei	pH	1	1.58	22.22	**<0.001**
	Light	1	0.71	10.00	**0.003**
	Flow	1	0.12	1.75	0.193
	pH x Light	1	0.57	7.98	**0.007**
	pH x Flow	1	0.11	1.55	0.220
	Light x Flow	1	0.00	0.07	0.799
	pH x Light x Flow	1	0.45	6.29	**0.016**
	Residuals	40	0.07		
S. durum	pH	1	0.033	27.56	**<0.001**
	Light	1	0.018	15.10	**<0.001**
	Flow	1	0.007	6.14	**0.019**
	pH x Light	1	0.004	3.17	0.085
	pH x Flow	1	0.001	0.78	0.385
	Light x Flow	1	0.001	0.43	0.519
	pH x Light x Flow	1	0.003	2.31	0.139
	Residuals	31	0.001		

Table S2. ANOVA table showing the effect of treatments on pH_{cf} of the corals *Plesiastrea versipora* and *Acropora yongei*, and the coralline alga *Sporolithon durum*.

Species	Effect	df	MS	F	p
P. versipora	pH	1	0.095	48.40	**<0.001**
	Light	1	0.025	13.16	**0.001**
	Flow	1	0.007	3.77	0.059
	pH x Light	1	0.001	0.43	0.514
	pH x Flow	1	0.000	0.00	0.965
	Light x Flow	1	0.008	4.15	**0.048**
	pH x Light x Flow	1	0.002	1.08	0.304
	Residuals	41	0.002		
A. yongei	pH	1	0.030	18.67	**<0.001**
	Light	1	0.000	0.00	0.953
	Flow	1	0.008	4.79	**0.034**
	pH x Light	1	0.001	0.47	0.497
	pH x Flow	1	0.010	6.23	**0.017**
	Light x Flow	1	0.000	0.24	0.625
	pH x Light x Flow	1	0.002	0.97	0.331
	Residuals	41	0.002		
S. durum	pH	1	0.130	62.97	**<0.001**
	Light	1	0.000	0.09	0.764
	Flow	1	0.003	1.84	0.183
	pH x Light	1	0.000	0.02	0.904
	pH x Flow	1	0.002	1.14	0.292
	Light x Flow	1	0.003	1.61	0.212
	pH x Light x Flow	1	0.000	0.05	0.829
	Residuals	37	0.002		

Table S3. ANOVA table showing the effect of treatments on the dissolved inorganic concentration in the calcifying fluid (DIC_{cf}) of the corals *Plesiastrea versipora* and *Acropora yongei*, and B/Ca (an indicator of dissolved inorganic concentration in the calcifying fluid) of the coralline alga *Sporolithon durum*..

Species	Effect	df	MS	F	p
P. versipora	pH	1	535180	17.29	**<0.001**
	Light	1	133715	4.32	**0.044**
	Flow	1	2873	0.09	0.762
	pH x Light	1	17552	0.57	0.456
	pH x Flow	1	58510	1.89	0.177
	Light x Flow	1	23455	0.76	0.389
	pH x Light x Flow	1	134121	4.33	**0.044**
	Residuals	41	30951		
A. yongei	pH	1	43047	9.79	**0.003**
	Light	1	8414	0.19	0.664
	Flow	1	5932	0.14	0.715
	pH x Light	1	83936	1.91	0.174
	pH x Flow	1	53948	1.23	0.274
	Light x Flow	1	16082	0.37	0.548
	pH x Light x Flow	1	112302	2.55	0.112
	Residuals	41	43946		
S. durum	pH	1	28	0.01	0.905
	Light	1	289	0.15	0.702
	Flow	1	8895	4.57	**0.039**
	pH x Light	1	126	0.07	0.800
	pH x Flow	1	7117	3.66	0.063
	Light x Flow	1	222	0.11	0.738
	pH x Light x Flow	1	0	0.00	0.992
	Residuals	37	1947		

Table S4. ANOVA table showing the effect of treatments on the aragonite saturation state in the calcifying fluid $\Omega_{arag\ cf}$ of the corals *Plesiastrea versipora* and *Acropora yongei*, and FWHM (an indicator of the calcite saturation state) of the coralline alga *Sporolithon durum*.

Species	Effect	df	MS	F	p
P. versipora	pH	1	0.106	0.50	0.484
	Light	1	0.081	0.38	0.541
	Flow	1	1.033	4.84	**0.034**
	pH x Light	1	0.013	0.06	0.805
	pH x Flow	1	0.122	0.57	0.453
	Light x Flow	1	0.114	0.54	0.468
	pH x Light x Flow	1	1.035	4.86	**0.033**
	Residuals	41	0.213		
A. yongei	pH	1	0.845	1.66	0.205
	Light	1	0.535	1.05	0.311
	Flow	1	1.122	2.21	0.145
	pH x Light	1	0.352	0.69	0.410
	pH x Flow	1	0.002	0.00	0.955
	Light x Flow	1	0.800	1.57	0.217
	pH x Light x Flow	1	0.004	0.01	0.935
	Residuals	41	0.509		
S. durum	pH	1	0.063	1.71	0.200
	Light	1	0.001	0.03	0.857
	Flow	1	0.056	1.52	0.225
	pH x Light	1	0.081	2.20	0.147
	pH x Flow	1	0.007	0.20	0.655
	Light x Flow	1	0.001	0.02	0.897
	pH x Light x Flow	1	0.000	0.00	0.985
	Residuals	37	0.037		

Table S5. ANOVA table showing the effect of treatments on Ca^{2+}_{cf} of the corals *Plesiastrea versipora* and *Acropora yongei*.

**Species**	**Effect**	**df**	**MS**	**F**	**p**
P. versipora	pH	1	6.455	20.63	**<0.001**
	Light	1	2.070	6.62	**0.014**
	Flow	1	0.273	0.87	0.356
	pH x Light	1	1.003	3.21	0.081
	pH x Flow	1	0.181	0.58	0.451
	Light x Flow	1	0.429	1.37	0.248
	pH x Light x Flow	1	0.293	0.94	0.339
	Residuals	41	0.313		
A. yongei	pH	1	0.225	0.205	0.653
	Light	1	0.085	0.077	0.783
	Flow	1	7.041	6.404	**0.015**
	pH x Light	1	0.750	0.682	0.414
	pH x Flow	1	1.105	1.005	0.322
	Light x Flow	1	0.588	0.535	0.469
	pH x Light x Flow	1	0.031	0.028	0.867
	Residuals	41	1.100		

Table S6. ANOVA table showing the effect of treatments on the photosynthetic rates of the corals *Acropora yongei* and *Plesiastrea versipora*, and the coralline alga *Sporolithon durum*.

Species	Effect	df	MS	F	p
A. yongei	pH	1	$0.17\ 10^{-5}$	0.01	0.93
	Light	1	$6.9\ 10^{-5}$	0.37	0.55
	Flow	1	$8.5\ 10^{-4}$	4.61	**0.042**
	pH x Light	1	$0.1\ 10^{-6}$	0.01	0.943
	pH x Flow	1	$1.4\ 10^{-4}$	0.77	0.399
	Light x Flow	1	$0.65\ 10^{-4}$	0.35	0.558
	pH x Light x Flow	1	$1.2\ 10^{-4}$	0.66	0.425
	Residuals	24	$1.8\ 10^{-4}$		
P. versipora	pH	1	$3.8\ 10^{-4}$	0.73	0.403
	Light	1	$2.4\ 10^{-3}$	4.58	**0.043**
	Flow	1	$1.1\ 10^{-4}$	0.22	0.645
	pH x Light	1	$6.9\ 10^{-4}$	1.31	0.265
	pH x Flow	1	$4.5\ 10^{-4}$	0.85	0.367
	Light x Flow	1	$1.2\ 10^{-4}$	0.22	0.642
	pH x Light x Flow	1	$2.4\ 10^{-5}$	0.05	0.834
	Residuals	23	$5.2\ 10^{-4}$		
S. durum	pH	1	$1.7\ 10^{-6}$	0.39	0.535
	Light	1	$2.0\ 10^{-6}$	0.46	0.504
	Flow	1	$8.0\ 10^{-9}$	0.00	0.966
	pH x Light	1	$6.6\ 10^{-6}$	1.51	0.231
	pH x Flow	1	$3.8\ 10^{-6}$	0.86	0.363
	Light x Flow	1	$2.0\ 10^{-6}$	0.47	0.501
	pH x Light x Flow	1	$2.8\ 10^{-8}$	0.01	0.937
	Residuals	24	$4.4\ 10^{-6}$		

Table S7. ANOVA table showing the effect of treatments on ΔpH DBL of the coral *P. versipora* and the coralline *S. durum*.

Species	Effect	df	MS	F	p
P. versipora	pH	1	0.067	4.48	**0.050**
	Light	1	0.322	21.64	**<0.001**
	Flow	1	0.192	12.91	**0.002**
	pH x Light	1	0.097	6.53	**0.021**
	pH x Flow	1	0.214	14.43	**0.002**
	Light x Flow	1	0.000	0.01	0.933
	pH x Light x Flow	1	0.012	0.79	0.387
	Residuals	16	0.238		
S. durum	pH	1	0.011	1.04	0.321
	Light	1	0.108	9.86	**0.006**
	Flow	1	0.025	2.29	0.149
	pH x Light	1	0.008	0.69	0.419
	pH x Flow	1	0.044	4.03	0.061
	Light x Flow	1	0.037	3.40	0.083
	pH x Light x Flow	1	0.009	0.77	0.392
	Residuals	17	0.011		
